# Patient Rounds With Video-Consulted Relatives: Qualitative Study on Possibilities and Barriers From the Perspective of Healthcare Providers

**DOI:** 10.2196/12584

**Published:** 2019-03-25

**Authors:** Christina Østervang, Lene Vedel Vestergaard, Karin Brochstedt Dieperink, Dorthe Boe Danbjørg

**Affiliations:** 1 Department of Oncology Odense University Hospital Odense Denmark; 2 The Danish Knowledge Centre for Rehabilitation and Palliative Care (REHPA) Odense University Hospital Nyborg Denmark; 3 Department of Clinical Research University of Southern Denmark Odense Denmark; 4 Department of Heamatology Odense University Hospital Odense Denmark; 5 Centre for Innovative Medical Technology Odense University Hospital Odense Denmark

**Keywords:** telehealth, family, relatives, cancer, technology, qualitative research

## Abstract

**Background:**

In cancer settings, relatives are often seen as a resource as they are able to support the patient and remember information during hospitalization. However, geographic distance to hospitals, work, and family obligations are reasons that may cause difficulties for relatives’ physical participation during hospitalization. This provided inspiration to uncover the possibility of telehealth care in connection with enabling participation by relatives during patient rounds. Telehealth is used advantageously in health care systems but is also at risk of failing during the implementation process because of, for instance, health care professionals’ resistance to change. Research on the implications for health care professionals in involving relatives’ participation through virtual presence during patient rounds is limited.

**Objective:**

This study aimed to investigate health care professionals’ experiences in using and implementing technology to involve relatives during video-consulted patient rounds.

**Methods:**

The design was a qualitative approach. Methods used were focus group interviews, short open interviews, and field observations of health care professionals working at a cancer department. The text material was analyzed using interpretative phenomenological analysis.

**Results:**

Field observational studies were conducted for 15 days, yielding 75 hours of observation. A total of 14 sessions of video-consulted patient rounds were observed and 15 pages of field notes written, along with 8 short open interviews with physicians, nurses, and staff from management. Moreover, 2 focus group interviews with 9 health care professionals were conducted.

Health care professionals experienced the use of technology as a way to facilitate involvement of the patient’s relatives, without them being physically present. Moreover, it raised questions about whether this way of conducting patient rounds could address the needs of both the patients and the relatives. Time, culture, and change of work routines were found to be the major barriers when implementing new technology involving relatives.

**Conclusions:**

This study identified a double change by introducing both new technology and virtual participation by relatives at the same time. The change had consequences on health care professionals’ work routines with regard to work load, culture, and organization because of the complexity in health care systems.

## Introduction

### Background

When a patient receives a cancer diagnosis, it will not only affect the patient but also the entire family surrounding the patient [1,2]. Relatives are often seen as a resource in connection with the course of the illness, both when the patients are at home and during admission to the hospital [1,3,4].

However, as Kahriman and Zaybak found in their study, relatives may feel that they are taking on a great responsibility of caring for the patient and all the practicalities related to the illness. As a result of that responsibility, relatives may experience a burden that can have an impact on their physical, emotional, and psychological health [5]. One way to support relatives is to educate them to support the patient [1,6,7]. A systematic review investigated how technology could be a support intervention for relatives of patients with cancer. It found that educational websites and smartphone apps had shown great potential to help ease the family’s burden and experiences during the patient’s trajectory [8]. A randomized controlled trial by Collinge et al evaluated a multimedia instructional program for family caregivers and found that by using technology both self-efficacy and satisfaction in caregiving was enhanced [9]. Furthermore, Fuentes et al tested a mobile system that maps relatives’ social network to prevent social isolation and found that the tool was perceived valuable [10]. However, the systematic review states that many technological solutions to support relatives already exist but minimal research focusing on improving active participation from relatives through a 2-way face-to-face communication has been published and is leaving a gap for further research [8]. During hospitalization, many patients request their relatives to actively participate when decisions about treatment and care are made, which often happens during patient rounds [11]. Relatives can support the patient by remembering and understanding the information given by nurses and physicians [3]. Direct involvement of the relatives may not only result in reducing the burden they feel, but their participation will also bring positive aspects for the patient by reducing frequency and length of hospitalizations [1,2,12]. However, work, family obligations, and distance to the hospital are reasons that cause difficulties for relatives to be present at the hospital and therefore complicate their participation in patient rounds [11,13]. Rising et al found that by using videoconferencing platforms, health care professionals were able to facilitate relative’s participation during patient rounds remotely [11]. This inspired us to look at the possibilities that telehealth care provides to enable increased relative participation for hospitalized patients.

Telehealth has been a priority in many countries for years and represents solutions that could provide some of the answers to the challenges that health care systems are facing [14]. These challenges include demographic changes with more elderly patients living with chronic conditions and long distances to hospitals [11,13-16]. In cancer care, the use of telehealth has, as mentioned above, shown beneficial aspects not only as support interventions for relatives but also as a positive addition to promote adherence in treatment and care, improving patient outcomes [17,18]. In spite of these experiences using telehealth, numerous barriers must be considered if the implementation is to succeed [14]. A systematic review shows that the barriers of implementation are often directly related to the culture among the health care professionals. Resistance to changes in working procedures along with their unwillingness to invest time in training in the new workflows were significant barriers [19]. According to Ross et al, potential barriers should be identified early in the process when planning implementation of changes. This will allow formulation of strategies to prevent resistance to change [20]. It is essential that there is a *sense of urgency* if the change is to be carried out among the health care professionals, in addition to a coalition of efficient people who can guide, coordinate, and communicate the change [21]. Although these factors are present, there is still a big risk of the implementation failing [19]. Many studies describe impacts and experiences of telehealth used as part of the treatment among patients and health care professionals [22-24], but in-depth research that explores the implications for health care professionals by involving relatives through a virtual presence is limited [11,13,15].

### Objective

The aim of this study was to investigate health care professionals’ experiences in using and implementing technology to involve relatives during video-consulted patient rounds.

## Methods

### Study Design

This is a qualitative study inspired by a phenomenological and hermeneutical position, investigating the experiences of health care professionals participating in video-consulted patient rounds with relatives. Qualitative research is characterized by collection, organization, and interpretation of textual data stemming from statements, conversation, and behavior [25]. The research design is in accordance with the consolidated criteria for reporting qualitative research appropriate for investigating the meanings of the social phenomena experienced and told by individuals, which in this study concerned video-consulted patient rounds [26]. The chosen methods are field observational studies, short open interviews, and focus groups. To obtain insight into the health care professionals’ perspective, interpretative phenomenological analysis (IPA; Textbox 1) is used for data analysis [27]. The data from the field observations made it possible to organize the focus group interviews using questions and tasks, related and relevant to specific observations made in connection with video-consulted patient rounds. The combination of the 2 methods also made it possible to strengthen the analysis by validating the field notes with quotes.

**An excerpt of the 6 steps in interpretative phenomenological analysis (IPA) from 1 case, showing the emergence of a main theme.**

Step 1: Reading and rereading

- Authors read each case several times

Step 2: Initial noting

- Describe frustrations about the arranged set of time
  - To be a slave of time
  - Time takes control
  - Have to get enough time to be well-prepared
  - A set of time is predetermined that is difficult

Step 3: Developing emergent themes

- Perspective of time
- To be prepared
- Structure of the day

Step 4: Searching for connections across emergent themes—superordinate themes

- Do not wish to be controlled by time

- Content and structure for the conversation

Step 5: Moving to next case

- Authors clear their minds before moving to the next case

Step 6: Looking for patterns across cases

- Just one more thing on the to-do list

### Setting and Technology

The study was conducted at a cancer department in the Region of Southern Denmark. The cancer department is a part of a large hospital with 42 departments and 8700 employees. The bed unit where the study was conducted treats 1300 patients per year. This study is part of a larger study, which also includes the perspective of relatives.

For this study, eligible patients were approached at the ward during admission by the first or second author to assess their interest in participating in video-consulted patient rounds. The patients were under active medical treatment owing to their cancer diagnosis and were aged from 61 to 86 years. They had been preselected by the staff nurse, nurses, and physicians at the department based on the inclusion criteria: patients able to hear, able to understand and speak Danish, who are expected to be hospitalized for more than 2 days, and who have relatives with internet connection to their smartphone, tablet, or personal computer.

The connection was provided with the use of the Cisco Jabber app. Staff at the hospital used an administration tool to set up a Cisco Jabber guest link for the relative. The relatives received the link by email. The link was connected to an ad hoc virtual meeting room, which meant that a link could be created for each relative. It required internet access and one of the following devices available to activate the link and participate in video-consulted patient rounds: personal computer, tablet, or smartphone. The staff used a Jabber unified communication client on a tablet. The video conversation was encrypted and complied with the security requirements in line with the Danish legislation concerning management of personal information. The relatives were educated in both oral and written formats on how to use the technology and were also provided with a support line.

### Recruitment

Participants were purposively recruited from the cancer department. Participants for the field observational study were nurses and physicians working at the cancer department, both those who carried out the video-consulted patient rounds and those who participated in discussions about it.

At the morning conferences, the attending nurses and physicians were orally informed about which patients had a video-consulted patient round arranged for that day. They were informed by the head nurse that either the first or the second author would be present for the purpose of observation. For the focus group interviews, the inclusion criteria were more rigorous. The inclusion criteria were as follows: health care professionals employed at the cancer department and registered nurses or physicians who had worked with video-consulted patient rounds with relatives once or several times. The exclusion criterion was as follows: physicians and nurses who were employed temporarily. In total, 12 participants were identified and asked by email if they would take part in one of the focus group interviews. Participants who agreed to participate were included in the focus group interviews, which were carried out in January 2018. Descriptive statistics such as professional background, experience, age, and gender were collected.

### Data Collection

Data collection was carried out by the first and second authors, both having many years of experience in nursing but no prior connection to the cancer department.

#### Field Observational Studies

Field observational studies were carried out for 3 days a week, for a total of 5 weeks from October 2017 to January 2018. The first and second authors were present at the department for 4 to 5 hours per day.

As described by Green and Thorogood, the field observational studies allowed us to directly obtain knowledge about what participants do and what they say they do, in connection with video-consulted patient rounds [28]. Furthermore, it provided the opportunity to conduct short open interviews in the field, posing a few open questions to physicians and nurses [29]. The questions were determined and verified as part of discussions among the research group and also based upon the field observations. The field observational studies were carried out with the acceptance of the department’s management. The nurses and physicians who were involved in the video-consulted patient rounds in different ways, such as planning, scheduling, and discussions of content in the actual consultations, were observed. The observations were carried out in 2 ways inspired by James Spradley’s description of *moderate participation* and *passive participation* [30]. *Moderate participation* was carried out as the first and second authors conducted observations of all sessions with video-consulted patient rounds. Before and after the patient rounds, both authors carried out small open interviews, gaining understanding of the participants’ thoughts and experiences in addition to the observations. In addition, *passive participation* was applied in relation to video-consulted patient rounds. First and second authors listened and observed the work of health care professionals by being present in the conference room, the hallway, the offices, and in the hospital rooms. As Spradley outlines, these observations gave firsthand knowledge of and insight into verbal and nonverbal statements and actions [30]. For each day, field notes were taken as keywords and were later in the same day transcribed into continuous text to secure correct recall [28].

#### Focus Groups

In addition to the field observational studies, focus groups were conducted in January 2018. We chose focus groups as they allow the researcher to obtain knowledge from the interactions between the participants, and we wanted to mobilize associations where the dynamics between the participants contribute to creation of narratives and discussions about the use of video-consulted patient rounds [28]. Focus groups were built around the specific topic, video-consulted patient rounds, and the interaction in the group facilitated discussions about the use of technology to enable the participation of relatives [28]. A semistructured interview guide with open questions and tasks guided the focus groups. Each focus group interview was split into 2 parts. First, the participants were asked to write down 3 positive and negative thoughts about video-consulted patient rounds with relatives and afterward they discussed their opinions. Second, participants were introduced to quotes spoken by relatives concerning video-consulted patient rounds and the following discussions started with the participants’ thoughts about the quotes.

Special effort was put into creating the groups, as groups that were too homogeneous risked a lack of interaction and groups that were too heterogeneous risked larger disagreements [31]. The first author facilitated the sessions located in a conference room at the hospital. The second author was present as an observer, writing field notes and validating the content of the discussion.

### Ethical Considerations

In accordance with the Helsinki Declaration and the Ethical Guidelines for Nursing Research in the Nordic countries, the participants were informed both orally and in writing, and we obtained written consent [32]. All participants received an information letter describing the aim and focus of the project. They were informed about their right to withdraw from the study, at any time, without consequences, and that their data would be anonymized. Furthermore, participants agreed to show respect and confidentiality about statements made during the focus group. Pictures taken during the sessions were only used with written consent from the participants. According to Danish legislation at the time of this study, the study did not need ethical approval or approval from the National Committee on Health Research Ethics. The study is registered with the Danish Data Protection Agency (17/43851). The data are stored in SharePoint (Microsoft Corporation).

### Data Analysis

In accordance with the qualitative research approach, the transcripts were analyzed using the detailed 6 step guide for IPA provided by Smith and Osborn [33] as shown in Textbox 1. IPA is phenomenological in that it concerns exploring experiences in its own terms and adds the ideographic and hermeneutical philosophy to interpret a small sample size [33].

Going through the initial focus group data and initial observational data required different processes as the interpretations were being derived from different positions. Data from focus group interviews emerged directly from the participant’s spoken words, whereas data from the field observations occurred as a product of the first and second authors’ observations and preunderstandings. Therefore, the first and second authors handled steps 1 to 4 in the IPA process separately for each dataset. This analytical process involved reading the transcripts several times, followed by open coding with a focus on the descriptive comments, leading to superordinate themes [33]. To ensure that the participants’ experiences were adequately represented in the themes, data were continuously checked throughout the entire process. The first and second author read the transcripts separately, followed by discussions to identify themes and codes in the data. From the list of superordinate themes, 3 main themes were derived from patterns across the focus group interviews combined with the field observations. Synthesizing the data at step 6 was considered to supplement and validate the results of the analysis by underlining equivalence and differences. To ensure identification of convergence and divergence in the data, themes were arranged using NVivo 11 (QSR International).

## Results

### Description of the Participants

In total, 12 health care professionals were approached and we obtained informed consent from 9 to participate in the focus group interviews. The 3 health care professionals who did not attend the focus groups had busy schedules. The population of participants consisted of 7 nurses and 2 physicians, 3 men and 6 women aged between 24 and 60 years, and their professional experience ranged between 6 months and 25 years (Table 1).

Field observational studies were conducted for a total of 15 days, which yielded a total of 75 hours of observation. A total of 8 short open interviews were carried out with physicians, nurses, and staff from the department’s management. In total, 14 sessions of video-consulted patient rounds were observed, and 15 pages of field notes written. On the basis of IPA, 3 main themes were derived from patterns across the focus group interviews, field notes, and transcripts of short open interviews. The themes are as follows (Table 2):

1. Relatives can qualify the conversation.
2. Is It a patient round or a family round?
3. Just one more thing on the to-do list.

### Relatives Can Qualify the Conversation

This theme was derived from consistent expressions by physicians and nurses concerning relatives’ positive impact during patient rounds, which was a universal assumption at the department. Health care professionals described the relatives’ involvement as a resource for obtaining useful information, helping the patient to remember important information and to follow up on, for example, changes in the medical treatment:

The physician talks about new medication, the patient looks at his daughter at the screen, he looks confused. The daughter says that she will pick it up at the pharmacy and bring it to his home after discharge.

Field note, December 13, 2017

Furthermore, health care professionals experienced great willingness by the relatives to participate and take responsibility for the information given at the patient rounds. Health care professionals experienced that using telehealth to enable participation by relatives reduced the number of misunderstandings during the hospitalization and reduced stress among both patients and relatives:

Many misunderstandings are reduced or prevented when relatives participate

Nurse, 26 years

The technology made it possible for the relatives to extend the conversation further by making corrections, for example, of what was realistic after discharge. In this context, health care professionals experienced that the visual contact was an improvement compared with a phone, because of the possibility to visualize the patient’s home:

The relative could walk around in the patient’s living room with the camera. That way we are able to see and direct focus on the challenges with for example mobilization after discharge.

Nurse, 43 years

The technology made it possible to deliver information related to treatment and care to the relative on the same day they were given to the patient, instead of waiting for several days until the relative could be physically present or reached by phone:

Specific details about the discharge are arranged, and the relative contributes with clarifying information, which the patient was not able to remember. The patient looks at her daughter at the screen and smiles, she looks relieved.

Field note, November 20, 2017

Health care professionals described how the technology made it possible to involve cross-sectional collaborators, for instance, rehabilitation homes, simultaneously with the participation of the relative:

The staff at the rehabilitation home was able to see him and talk to him. Moreover, they met his wife virtually and talked to her. It sounded like some of the wife’s concerns disappeared.

Nurse, 54 years

Health care professionals experienced that this way of preparing discharges could minimize anxiety related to the discharge for both the patient and the relative.

### Is it a Patient Round or a Family Round?

In general, health care professionals experienced video consultation as a possible way of accommodating participation by both patients and relatives in treatment and care.

This is a good way of getting knowledge about the relatives too.

Short open interview with manager, January 6, 2018

Although the health care professionals recognized the opportunities in involving both relatives and patients in the consultation, they also emphasized that they unintentionally directed their focus toward the tablet screen and indirectly the relative, rather than focusing on the patient. They did not feel comfortable using the new technology, mostly because they felt challenged by the amount of attention they had to direct at the relative behind the screen:

I think that it’s difficult to turn my focus away from the screen, suddenly you are at risk of forgetting the patient. It’s important for me that we talk directly to the patient… but of course we should listen to the relative too.

Nurse, 26 years

**Table 1:** Sociodemographics of health care professionals (n=9) in a Danish study of video-consulted patient rounds at a cancer department.

Sociodemographics		Statistics
**Profession, n**		
	Nurse	7
	Physician	2
**Age (years)^a^** **, mean**		
	Nurse	37
	Physician	46
**Professional experience (years)^b^**		
	<5 years	4
	>5 years	5
Number of participations in patient rounds, mean		2

^a^Range: 24 to 60 years.

^b^Range: 4 months to 22 years.

**Table 2:** From superordinate themes to 3 main themes in a Danish study investigating telehealth in patient rounds.

Examples of superordinate themes		Main themes
**First theme**		
	Easy access to information	Relatives can qualify the conversation
	Recognizable technology	Relatives can qualify the conversation
	Reduce misunderstandings	Relatives can qualify the conversation
**Second theme**		
	Content of the conversation is important	Is it a patient round or a family round?
	Expectations from the relatives	Is it a patient round or a family round?
	The presence of the screen	Is it a patient round or a family round?
**Third theme**		
	Being controlled by time is stressful	Just one more thing on the to-do list!
	New workflows are difficult	Just one more thing on the to-do list!
	High workload	Just one more thing on the to-do list!

In relation to the screen, the health care professionals stressed that it would require a period of time to learn how to use the technology, which they perceived as a new coplayer. All participants agreed that the definition of the content in the conversation had great importance. That way they were able to establish a regulatory framework that still made it possible for them to plan their work, in spite of this new way to do patient rounds:

This is new, and we have to learn to use it, and to define the content of the conversation. We also have to acknowledge that the rounds are held with focus on the patient.

Physician, 43 years

The health care professionals expressed the importance of matching expectations with relatives before their participation. This way, it would be clearly stated what the health care professionals were able to offer.

Health care professionals found that the solution was time-consuming and that it could result in them taking care of the needs expressed by relatives too, which they did not find themselves capable of:

The conference room is filled; an interdisciplinary meeting is held. Some are standing up. The discussion is concerning the selection of patients to participate in video consulted patient rounds. One physician says: this way of doing patient rounds only takes the relatives into account. Not the patients. Another physician says, it could be time-consuming involving the family. Nurses and managerial staff are silent.

Field note, December 19, 2017

Nurses and physicians experienced that for the video-consulted patient rounds to be a success, they must have a clearly defined structure and definition of content in the conversation, which everybody in the department agrees to follow.

### Just One More Thing on the To-Do List

The majority of the health care professionals found the technology easy to use and recognizable from their everyday lives, using FaceTime or Skype. However, when image or sound quality failed one or more times during some sessions, it felt stressful and caused inconvenient disruptions to the conversation.

These interruptions were time-consuming and required health care professionals to have technical skills to be able to resolve the issues. This was a contributory reason for not arranging this type of patient rounds often.

Even though the technical solution is easy... The fact that I know from the beginning of the day that I’m responsible for the preparations - it’s just one more thing to do in a busy schedule.

Nurse, 25 years

Video-consulted patient rounds required a new way of organizing work, which was perceived as difficult and resistance to change was seen in more situations:

A health care professional says at the morning conference, that it would be nice if work was like in the old days. There’s silence in the room. The health care professional continues; “in the old days I only had to do patient rounds, not all these new things”. The majority of the health care professionals nod their heads, the managerial staff too.

Field note, November 20, 2017

The nurses experienced a great responsibility for the technical setup to be ready at the agreed time. The responsibility was not only for the tablet to be ready but also to ensure that the patient was ready, the relative had appeared live on the screen, and let the physicians know that everything was well-prepared:

You feel a great responsibility for everything to be ready, you don’t want to waste anyone’s time by them having to wait for you

Nurse, 25 years

Health care professionals also expressed that if a consistent work structure regarding the video-consulted patient rounds was established, it could free up time. They predicted that it could reduce the number of phone calls from relatives in the evening shift and this would result in freeing up more time to care for the patient:

Because relatives can participate and therefore inform the whole family, it could save the nurse on the evening shift many phone calls, where she retells the written journal text from earlier that day.

Nurse, 60 years

In both focus groups, health care professionals stressed that changing workflows was difficult because of their already high workload and in spite of introducing an *easy-to-use technology*, it required them to prioritize their assignments differently during the day.

## Discussion

### Principal Findings

This study identified a number of possibilities and barriers related to adopting a telehealth solution for including relatives. The overall finding was that health care professionals experienced the technology as a way to facilitate involvement of the patient’s family, although the relatives were not physically present. Moreover, it raised questions about whether this way of doing patient rounds could embrace the needs of both the patients and relatives. Time and change of work routines involving new technology were recognized as major barriers when implementing new technology involving relatives.

### Family-Centered Care

One of this study’s main findings was health care professionals agreeing on the positive impact of relatives participating in the patient rounds, which they experienced as a qualification of the conversation. Their virtual presence was able to reduce misunderstandings in the information given about treatment and care, benefitting the patient, the family, and the health care professionals. This is consistent with a study concluding that by having the family join the patient rounds via telemedicine, resolutions of an issue where family input is very important can be expedited [16]. However, another finding in our study showed that health care professionals discussed where the focus should lie, with the patient or with the relative. This statement was met with different attitudes. Some considered the relative equal to the patient, allowing them to take active part in the conversation, whereas others prioritized the perspective of the patient. In line with these findings, a systematic review found that family-centered decisions are highly dependent on how health care professionals recognize the patient and the family as one unit of care, in addition to the communication and attitudes used in their presence [34]. The benefits of using video-consulted patient rounds were the possibilities of providing more family-centered care connected to an increasing understanding of the whole family, although the levels of support by health care professionals varied [13]. Initiation of family participation in patient rounds is often based on the culture and tendencies of the team doing the patient rounds. Focus on the best way of communicating as part of the culture among health care professionals is of great importance in relation to optimizing family participation [35]. In our study, the discussion regarding involving relatives virtually raised diverse topics and attitudes. On one hand, it was experienced as important and useful in treatment and care but, on the other hand, it raised a debate of how much influence the family should have during the patient rounds. Health care professionals stressed that, in future, this would require an agreement on and matching expectations of how to provide family-centered care.

### Workload

In this study, both nurses and physicians found the technology easy to use; however, their workload played a significant role and, in some cases, seemed to overshadow the benefits of using the technology. They experienced it as *just one more thing* they had to offer in an already busy schedule. Moreover, they found that this solution was time-consuming. It required them to prioritize time to learn the new technology, attend the patient rounds at a predetermined time, and invest time in involving the relatives. These findings are compatible with the findings in a scoping review, which yielded 74 studies of adopting electronic health [14]. Lack of time and intense workload were significant barriers to the implementation of telehealth solutions. Telehealth was believed to take health care professionals away from well-known clinical tasks and place a greater workload onto them, which resulted in resistance to adapt [14]. Several studies argue that endorsement and clear visions from management are necessary if the staff is to be willing to invest extra time and effort in implementing new technologies [11,13]. In this study, the use of technology required a change in the existing work organization of the patient rounds for the health care professionals. This was due to the need for a specifically set time for doing patient rounds and also the involvement of relatives in the conversation. This indicates that many factors can have an impact on the success of technological information systems and that experiences of heavy workload and lack of time affect the possibility of adopting new work routines.

### Organization

Our findings revealed that health care professionals experienced organizational changes when faced with implementation of the new technology. They were *controlled* by a prearranged time for attending the patient rounds, combined with different professions, depending on each other to be present at that time. Therefore, accommodating the family perspective was experienced as overruling their rights to organize their own work. It left them reluctant to adapt to the implementation of the technology. Mohammadzadeh et al aimed to explain key considerations in telehealth solutions relating to cancer care. They found that the organizational structure and management provide an environment that can have a strong influence on the adoption of new technology. The organizational culture among health care professionals must contain acceptance of the need to implement the technology [36]. A sense of urgency among employees is the first and most crucial step when transforming organizations and making changes happen [21]. If the staff does not feel a sense of urgency, changes are most likely to fail. To prevent that from happening, a group with enough commitment to lead the change through a clear vision is essential for the implementation to succeed [21]. This is also supported by Rising et al, who suggest the presence of clinician champions in the department to take care of concerns raised by late or nonadopters [11].

### Double Change

**Figure 1:**
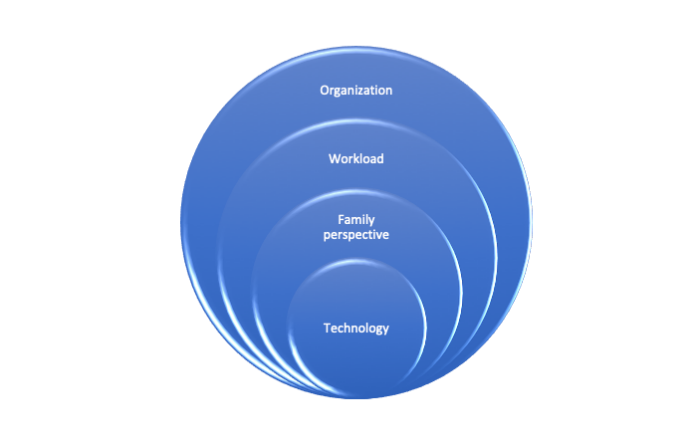
Illustration of the complexity in health care systems.

Furthermore, the study discovered that health care professionals experienced the technology as easy to use, but the screen seemed to draw their attention and they would start focusing on the relative instead of the patient. They described an experience of indirectly having to choose between the patient and the relative and allowed the screen to affect them in that way. This was due to the presence of the relative through the screen that highlighted the relative and suppressed the focus on the patient. According to Ihde, technology does nothing in itself, but in the interaction with human beings, the technology can, as in our study, *highlight* the relative as the contact is mediated through technology [37]. Technologies are mediators of human experiences and practices, where a human’s behavior and attitude toward the technology will shape its usage patterns [38]. With this understanding, the technology in our study only represents a solution to facilitate a conversation, but the use of the technology was influenced and dependent on the perception of health care professionals who attended the patient rounds. The health care professionals emphasized that it required time to learn to use a new communication tool. This is in line with what Danbjørg et al found in their study of nurses’ experiences of using a new app to support parents after early discharge. They found that the nurses needed time to adapt to new ways of communicating when technology is involved. With time, they enhanced possibilities not only in using the technology as a communication tool but also in allowing observations and emotional support [39]. In this study the technology itself seemed to be the smallest part but the health care professionals experienced changes when adopting the new technical skills required for handling the technology. Changes were also observed as health care professionals had to rethink the way they usually interacted with relatives, and heavy workloads seemed to influence the amount of resistance that occurred when adopting the family-orientated telehealth solution. These factors required an organizational change for both nurses and physicians, affecting their previous possibilities to organize their own working day. What was expected as implementation of an *easy-to-use technology* became an extremely complex process, leading us to think that the complexity of the health care organizations cannot be ignored. It tells us that changing one thing will often bring along many changes, which might end up having extensive consequences for health care professionals in clinical practice. Many of these impacts are difficult to predict, which leads us to the introduction of the following figure (Figure 1). The figure illustrates elements regarding the complexity of implementing new technology.

### Limitations and Strength

Our study is limited in using IPA as a research approach, because it requires that all the participants involved possess the necessary language as a tool to explain their experiences. Therefore, limitations in using IPA rely upon the validity of language. A small sample size often raises questions concerning representativeness and transferability of findings. Nevertheless, in IPA research, the aim is not to investigate what occurs in *all* settings, but to instead focus on the perceptions and understandings of a specific group within *their* setting [33,40]. Including participants who are colleagues could have affected the discussions by some participants, suppressing their opinions because of the internal dynamics among them. Both focus groups allowed every participant speaking time, and they all contributed in the interactions, which was the intended advantage of using focus groups. All quotes used in the Results section were member-checked by the individual participants, as a technique to improve accuracy and validity [41]. Mixing the methods and focus group interviews, as well as field observational studies, are considered the strength of this study. It allows statements from health care professionals to be supported and validated by observations leading to a broader understanding of the investigated phenomenon. This study also provided knowledge in an area with limited in-depth research—the use of telehealth with relatives in patient rounds.

### Conclusions and Implications for Clinical Practice

In conclusion, this study illustrated that telehealth in relation to relatives is experienced by health care professionals as a possible way to facilitate participation of the patient’s relatives in patient rounds. Their virtual presence at patient rounds reduces misunderstandings and improves decisions about treatment and care. It became clear that introducing telehealth with relatives required changes making an impact on health care professionals in more ways than one. We identified a double change, not only a change in using technology but also a change that had consequences for the health care professionals’ work routines in connection with workload, culture, and organization.

This study gave insight into the implementation of telehealth involving relatives that relies upon many factors because of the complexity in the health care organization. We learned that changes must be planned carefully into the existing organization structures, as a small change affects changes on many levels across the organization. Moreover, telehealth with relatives improves access to health care and quality in patient rounds because it may empower patients and relatives by providing more individualized information, treatment, and care.

## Abbreviations

IPA: interpretative phenomenological analysis
